# Desmoglein-4 Deficiency Exacerbates Psoriasiform Dermatitis in Rats While Psoriasis Patients Displayed a Decreased Gene Expression of DSG4

**DOI:** 10.3389/fimmu.2021.625617

**Published:** 2021-04-29

**Authors:** Tamara Moreno-Sosa, María Belén Sánchez, Elisa Olivia Pietrobon, Juan Manuel Fernandez-Muñoz, Felipe Carlos Martín Zoppino, Flavia Judith Neira, María José Germanó, Diego Esteban Cargnelutti, Alicia Carolina Innocenti, Graciela Alma Jahn, Susana Ruth Valdez, Juan Pablo Mackern-Oberti

**Affiliations:** ^1^Instituto de Medicina y Biología Experimental de Cuyo CONICET, Universidad Nacional de Cuyo, Mendoza, Argentina; ^2^Instituto de Histología y Embriología de Mendoza, Facultad de Ciencias Médicas, Universidad Nacional de Cuyo, Mendoza, Argentina; ^3^Hospital Luis Lagomaggiore, Mendoza, Argentina; ^4^Facultad de Ciencias Exactas y Naturales, Universidad Nacional de Cuyo, Mendoza, Argentina; ^5^Instituto de Fisiología, Facultad de Ciencias Médicas, Universidad Nacional de Cuyo, Mendoza, Argentina

**Keywords:** psoriasis, desmoglein, T cell, inflammation, RNAseq analysis

## Abstract

Desmogleins are involved in cell adhesion conferring structural skin integrity. However, their role in inflammation has been barely studied, and whether desmoglein-4 modulates psoriasis lesions is completely unknown. In this study, we assessed the impact of desmoglein-4 deficiency on the severity of imiquimod (IMQ)-induced skin inflammation and psoriasiform lesions. To this end, desmoglein-4^−/−^ Oncins France Colony A (OFA) with Sprague–Dawley (SD) genetic background were used. Additionally, human RNA-Seq datasets from psoriasis (PSO), atopic dermatitis (AD), and a healthy cohort were analyzed to obtain a desmosome gene expression overview. OFA rats displayed an intense skin inflammation while SD showed only mild inflammatory changes after IMQ treatment. We found that IMQ treatment increased CD3^+^ T cells in skin from both OFA and SD, being higher in desmoglein-4-deficient rats. In-depth transcriptomic analysis determined that PSO displayed twofold less DSG4 expression than healthy samples while both, PSO and AD showed more than three-fold change expression of DSG3 and DSC2 genes. Although underlying mechanisms are still unknown, these results suggest that the lack of desmoglein-4 may contribute to immune-mediated skin disease progression, promoting leukocyte recruitment to skin. Although further research is needed, targeting desmoglein-4 could have a potential impact on designing new biomarkers for skin diseases.

## Introduction

The desmosome is an intercellular junction that is crucial to support tissue integrity and cooperate in keeping tissue homeostasis (1). Desmogleins (Dsgs) are cadherin-type transmembrane molecules located in desmosomes and mainly involved in adhesion mechanisms (2). In skin, Dsgs are differentially expressed as basal cells differentiate into terminal keratinocytes (KC) from epidermis (3, 4). Dsg-2 and Dsg-3 are mainly expressed at lower skin epithelial layers while Dsg-1 is expressed in the upper layers (3). Dsg-4 is highly expressed in the hair follicle of human, mice, and rats (5–7). In addition, Dsg-4 is also expressed in the granular layer of the human epidermis, while in mice and rats, this location remains unclear (6). Surprisingly, it has been reported that loss-of-function alterations in Dsg-2 and Dsg-3 resulted in a deregulated proliferation and aberrant differentiation impairing tissue integrity (8, 9). Dsg-4 gene (DSG4) and protein sequences are conserved between human and other mammals, and its deficiency is clearly associated with sharp hair loss in human, mice, and rats, indicating an analogous function (5, 6, 10–13). Intracellular desmoglein domains interact with keratin filaments via plakoglobin, plakophilins, and desmoplakin, while extradesmosomal Dsg domains may interact with actin (14).

Psoriasis (PSO) pathogenesis can be attributed to the interaction between dendritic cells (DCs), T cells, and KCs with remarkable amounts of inflammatory cytokines (15–18). Strikingly, although most of our knowledge about Dsg functions concerns KC biology, regulation of the immune response by Dsgs has barely been addressed. Therefore, determining whether Dsg-4 is relevant in skin inflammation will provide further insight into the pathogenesis of PSO and immune mediated skin diseases as well.

The focus of this work was to evaluate the role of Dsg-4 in modulating skin inflammation following administration of topical imiquimod (IMQ), a Toll Like Receptor 7 (TLR7) ligand, which is experimentally used to emulate a psoriasis-like dermatitis (19, 20). For this, we used Dsg-4-deficient rats (Oncin's France Colony A/OFA), which bear a large intragenic deletion of the Dsg-4 gene encompassing nine exons and lack, or have very little, body hair when adult, with normal epidermis thickness and keratinization, and hyperthophic sebaceous glands (21, 22). We show that Dsg-4-deficient rats developed a more severe inflammatory phenotype with sharp hyperkeratosis following topical IMQ administration than Sprague–Dawley (SD) rats. Additionally, by transcriptomic analysis of human RNA-Seq datasets, we found that DSG4 mRNA levels was diminished in PSO samples. These results indicate that Dsg-4 collaborates in keeping IMQ-induced inflammation down.

## Results

### Desmoglein 4 Deficiency Exacerbates Inflammation

**Figure 1:**
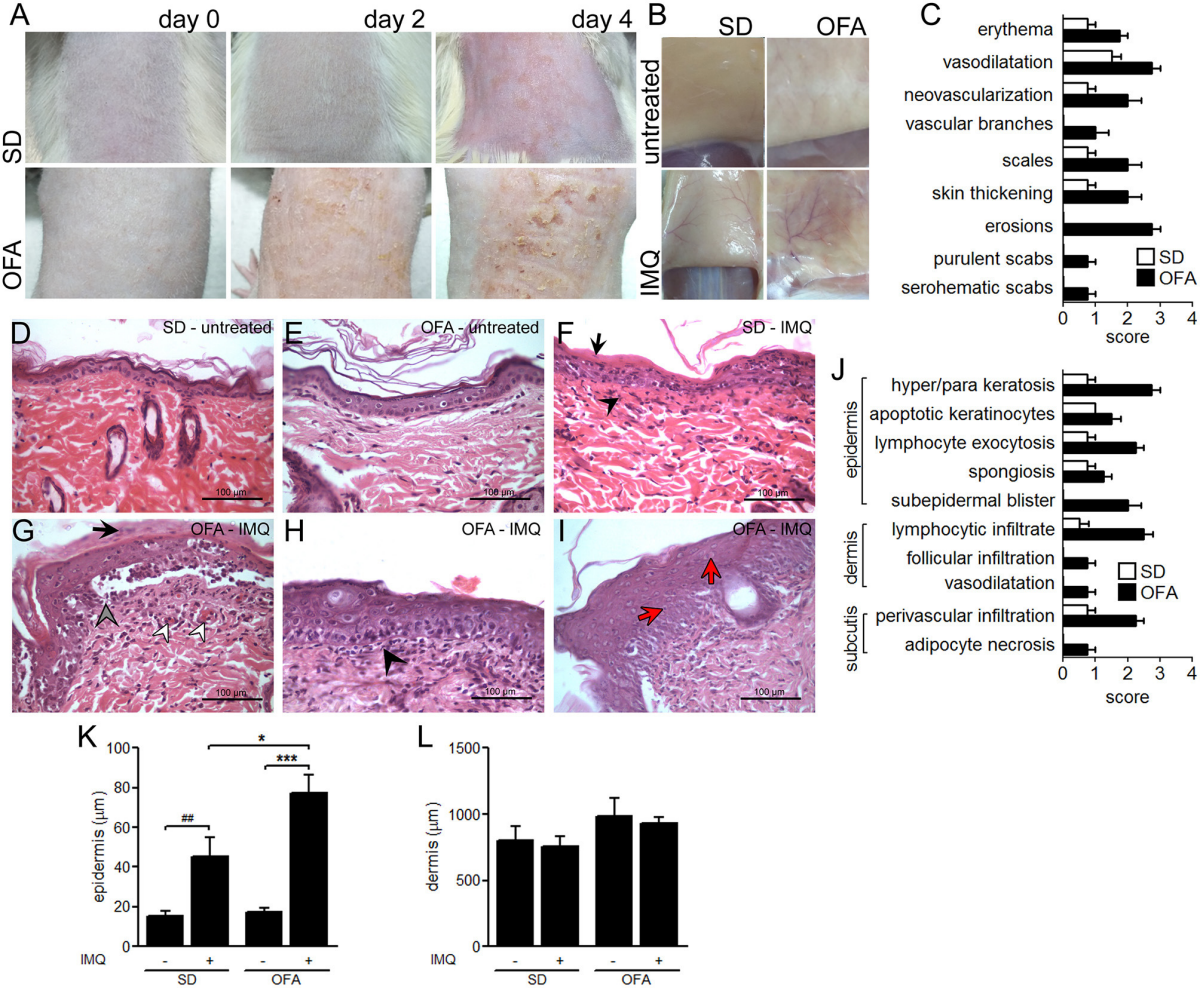
Psoriasiform-like lesion in OFA rats. **(A)** Evolution of IMQ-induced inflammation (days 0, 2, and 4). **(B)** Immediately after euthanasia on day 4, the skin area corresponding to the treated dorsal-lumbar zone was surgically pulled up and photographed by its inner side before tissue excision. **(C)** Clinical score. H&E histological studies on day 4 in untreated skin from SD **(D)** and OFA **(E)**, and IMQ-treated skin from SD **(F)** and OFA **(G–I)** rats. **(F)** Black arrows indicate parakeratosis, and black arrowheads denote interstitial mononuclear inflammatory infiltrate; **(G)** black arrows indicate parakeratosis, gray arrowhead indicates intense hydropic degeneration of basal cells, and white arrowheads denote vascular proliferation; **(H)** black arrowhead indicates interstitial mononuclear inflammatory infiltrate; **(I)** red arrows indicate dyskeratosis. **(J)** Histological scoring. **(K,L)** Thickness quantification of epidermis and dermis. (*n*_rats_/group = 6; three independent experiments). *P < 0.05, ***P < 0.001 with ANOVA and Bonferroni *post hoc* test was used. ^*##*^*P* < 0.01 *t* test between SD and IMQ-SD datasets.

To evaluate the role of Dsg-4 in the skin immune response, we induced acute skin inflammation in OFA and wild-type SD rats by topical administration of IMQ. IMQ-treated OFA rats displayed a worse clinical outcome with marked skin erythema, thicker skin, erosions, mild scabs (serohematic and purulent) and scaling, while IMQ-treated SD rats showed only scarce erythema with mild thickening (Figures 1A,C). Similarly, IMQ-treated Dsg-4-deficient rats displayed a striking dermal vascular response with neovascularization, vascular branching, and vessel enlargement compared to IMQ-treated SD rats (Figure 1B). Due to the possibility that Dsg4 deficiency causes an unspecific barrier breakdown that may facilitate infection and secondary inflammation, we evaluate the microbiological status of the IMQ-treated and untreated skin zones. Although IMQ treatment enhanced clinical lesions, bacterial loads consisting only in *Staphylococcus epidermis* were similar in all groups (Supplementary Figure 1A). Similarly, no compatible elements with fungi were observed in all samples analyzed (Supplementary Figures 1B–E). Thus, Dsg-4 deficiency exacerbates the inflammatory response as well as psoriasiform lesions after topical IMQ treatment without impact in the local microbiota.

### IMQ Induces Epidermal Disturbances in OFA Rats

To determine whether Dsg-4 plays a role in keeping epidermal homeostasis during an inflammatory response, we performed histological studies on IMQ-treated skin from OFA and SD rats. The lack of Dsg-4 interferes with the epithelial response to IMQ as evidenced by severe hyperkeratosis and parakeratosis, intense hydropic degeneration of basal cells, and marked vascular proliferation (Figures 1D,E,G–J). In contrast, IMQ-treated SD rats displayed only mild parakeratosis and slight vascular proliferation (Figure 1F). Similarly, epidermis thickness was more intense in OFA than in SD rats after IMQ treatment while dermis remained similar in all groups (Figures 1K,L). Although IMQ-treated OFA samples displayed changes in skin architecture, E-, N-, and VE-cadherin mRNA expression showed no changes (data not shown). Additionally, Dsg-4-deficient rats showed a differential inflammatory response to IMQ compared to SD rats consisting mainly of intense exocytosis of lymphocytes and neutrophils into the epidermis and marked interstitial mononuclear inflammatory infiltrate in the dermis (Figures 1F,H). In contrast, IMQ-treated SD rats only evidenced mild immune cell exocytosis in the epidermis and slight mononuclear infiltrate in the dermis (Figure 1F). The finding that Dsg-4 deficient rats displayed a more severe disruption and intense inflammation when compared with SD rats in response to IMQ indicates that this molecule would be involved in keeping skin homeostasis.

### Desmoglein 4 Deficiency Enhances the Expression of Inflammatory Genes in Response to IMQ

**Figure 2:**
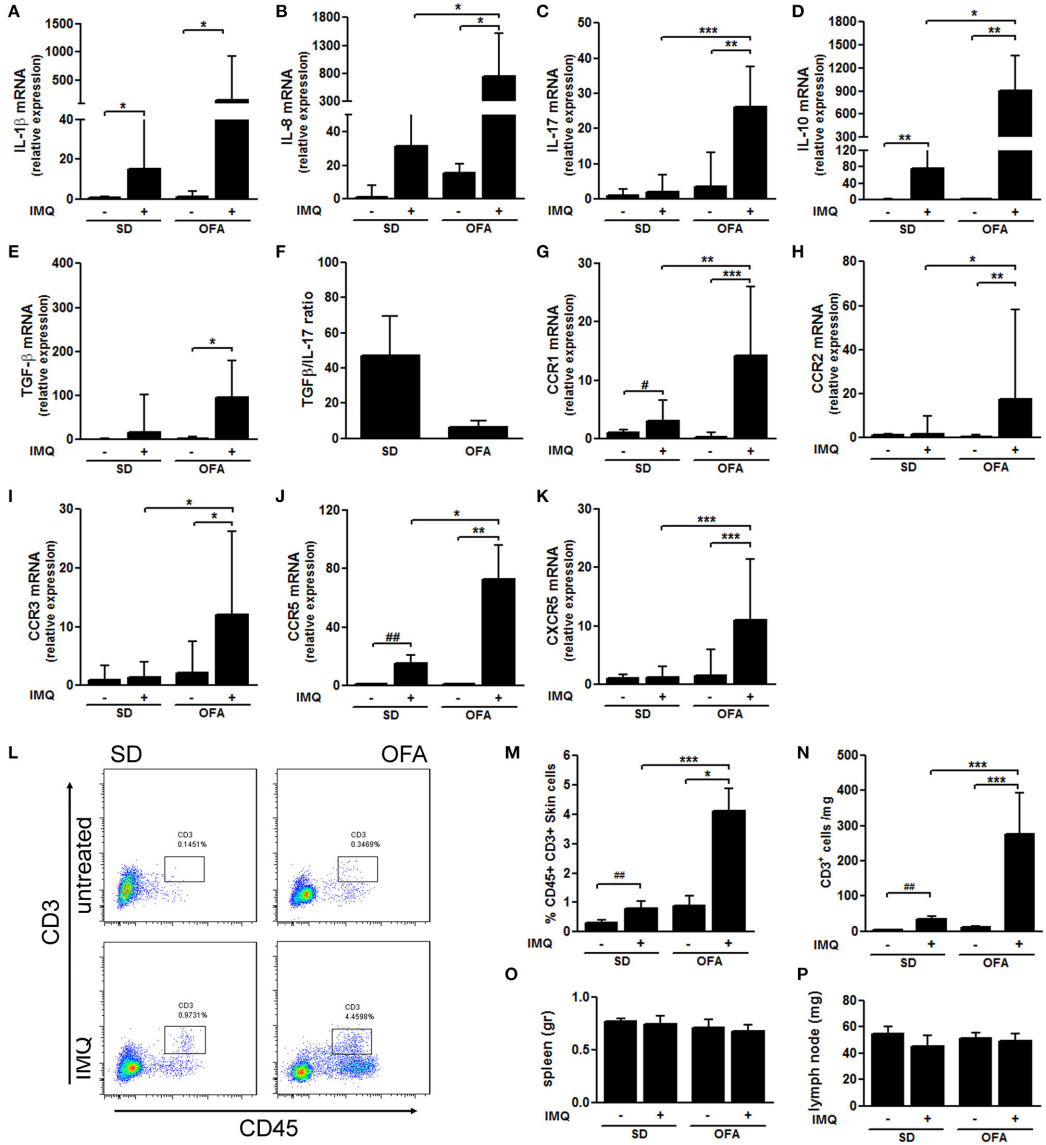
Dsg-4 deficiency induces high mRNA expression of inflammatory genes in response to IMQ. RNA from skin was extracted, and subsequently real-time PCR and cDNA synthesis were performed to evaluate mRNA abundance of cytokines: IL-1β **(A)**, IL-8 **(B)**, IL-17 **(C)**, IL-10 **(D)**, and TGF-β **(E)** performing Kruskal–Wallis tests analysis. Additionally, TGF-β/IL-17 mRNA abundance means ratio was calculated **(F)**. Similarly, chemokine receptors mRNA levels were measured: CCR1 **(G)**, CCR2 **(H)**, CCR3 **(I)**, CCR5 **(J)**, and CXCR5 **(K)**. Kruskal–Wallis and ANOVA tests were used; data represent the median with values range; *P < 0.05, **P < 0.01, ***P < 0.001. ^#^*P* < 0.05, ^*##*^*P* < 0.01 *t*-test between SD and IMQ-SD datasets. **(L)** Pseudocolor dot plots represent counts of infiltrating CD45^+^ cells and CD3^+^ cells from total skin cells analyzed by flow cytometry derived from SD and OFA rats. **(M,N)** Graphs represent the percentage and absolute cell counts of CD45^+^ CD3^+^ cells from total skin cells. (*n*_rats_/group = 6; two independent experiments). **(O,P)** Spleen and inguinal lymph node weights. FACS and lymphatic tissue weight data are represented as the mean ± SEM; *P < 0.05, **P < 0.01, ***P < 0.001 with ANOVA and Bonferroni *post hoc* test was used. ^#^*P* < 0.05, ^*##*^*P* < 0.01 *t*-test between SD and IMQ-SD datasets.

To identify immune mediators involved in the exacerbated IMQ-induced response observed in Dsg-4-deficient rats, we evaluated the expression of mRNA master cytokines in skin lesions by qPCR. We found that both OFA and SD rats increased mRNA expression of the pro-inflammatory cytokines IL-1β and IL-8 after IMQ treatment (Figures 2A,B); however, the deficiency of Dsg-4 induced higher mRNA levels. Surprisingly, when we evaluated IL-17 mRNA levels, we found that only OFA rats treated with IMQ increased its expression (Figure 2C). When we evaluated mRNA expression of two master anti-inflammatory cytokines, IL-10 and TGF-β, IMQ-treated OFA rats displayed a several-fold higher expression of IL-10 than the IMQ-treated SD group, but no significant differences in TGF-β response between both strains were observed (Figures 2D,E). Furthermore, when we determined the expression ratio between anti- and pro-inflammatory cytokines, we found that Dsg-4-deficient rats displayed a decreased TGF-β/IL-17 ratio (Figure 2F).

Similarly to what we observed in cytokine mRNA expression, we found that the lack of Dsg-4 potentiates the mRNA chemokine receptor profile induced by IMQ in SD rats. CCR1 and CCR5 genes displayed an increased mRNA expression in response to IMQ in both rat strains (Figures 2G,J). However, IMQ-treated OFA rats evidenced higher levels of these receptors than SD rats. In contrast, mRNA levels of CCR2, CCR3, and CXCR5 were only increased in skin from IMQ-treated OFA rats while the treatment failed to increase mRNA levels in SD rats (Figures 2H,I,K). These findings suggest that Dsg-4 keeps the infiltration of inflammatory cells to skin in response to IMQ controlled by differential expression of cytokines and chemokines.

In order to determine whether Dsg-4 restrains IMQ-induced T cell infiltration, we performed flow cytometry assays in inflamed skin samples from SD and OFA rats. Interestingly, we found that skin leukocyte population was slightly higher in untreated Dsg-4-deficient rats compared with SD, and they also displayed higher CD3^+^ T cell counts (Figures 2L,N). Similarly as observed in former histological studies, we found that leukocyte and CD3^+^ T cell infiltration in response to IMQ were enhanced in OFA rats compared to IMQ-treated SD rats (Figures 2L–N). In order to evaluate whether IMQ induced systemic inflammation, we measured spleen and inguinal lymph node weights resulting in no changes (Figures 2O,P). The finding that Dsg-4 deficiency increases T cell recruitment in response to IMQ suggests that Dsg-4 is involved in keeping lymphocyte migration to inflamed skin controlled.

### DSG4 mRNA Was Decreased in PSO

**Figure 3:**
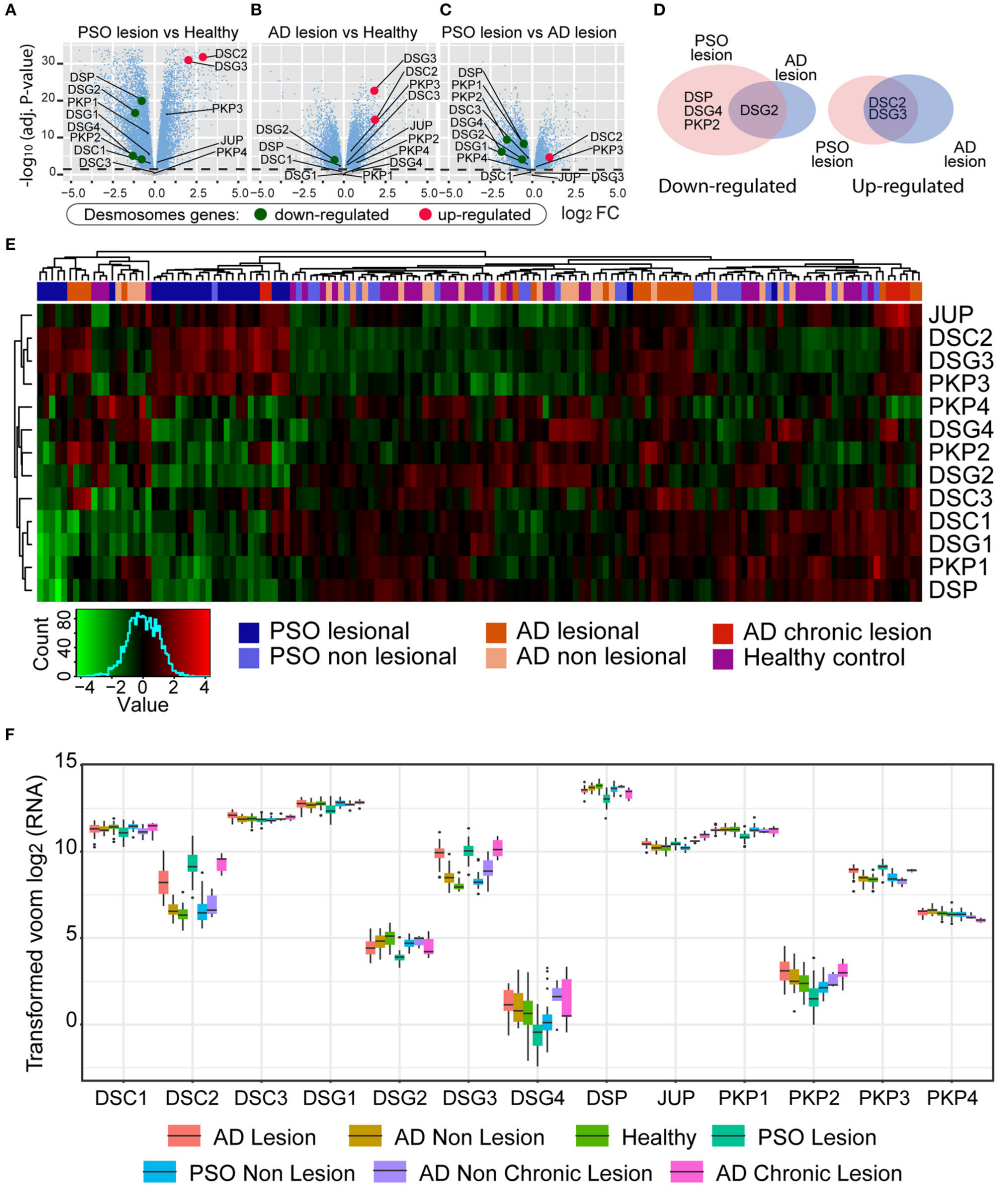
Differential gene expression (DGE) analysis achieved by *limma* R method. In the *y*-axis, –log_10_(adjust *P*-value), and in the *x*-axis, the log_2_ fold change of **(A)** PSO lesion vs. Healthy; **(B)** AD lesion vs. Healthy; **(C)** PSO lesion vs. AD lesion. **(D)** Venn diagram of significative deregulated (<0.5 and >2). **(E)** Gene expression heatmap of AD, PSO, and healthy skin samples. Expression patterns of desmosome genes are represented (low expression levels in green and high expression levels in red). **(F)** Gene expression boxplot of desmosome genes. Each color represents one of the seven conditions of the skin samples (AD lesions and non-lesions, Healthy skin, PSO lesions and non-lesions, and AD chronic lesions and non-chronic lesions).

To determine whether a decreased Dsg-4 level is associated with inflammatory skin diseases, we evaluated differential expressed genes (DEG) in PSO, AD, and healthy controls using available datasets (23). In this study, we considered only genes that showed absolute values of log_2_ fold change (log_2_FC) > 1 and statistical significance (FDR < 0.05). Surprisingly, when genes closely associated to desmosomes (Supplementary Tables 1, 2) were analyzed, only two of 13 genes, DSG3 and DSC2, were upregulated twofold or more in acute and chronic lesions of AD and PSO patients (Figures 3A,B,D and Supplementary Figure 2C) (2). In contrast, DSG4, DSP, and PKP2 mRNA expression were decreased to less than half of healthy exclusively in PSO while DSG2 was similarly lowered in both diseases (Figures 3A,B,D). When we compared PSO vs. AD to evaluate desmosome profiling similarities in inflammatory skin diseases, we found that DSG4, DSP, DSG2, and PKP2 displayed less than a two-fold difference in gene expression. Interestingly, DSC2 was the only gene with higher expression in PSO compared to AD lesions (Figures 3C,D). AD and PSO samples from non-lesional sites displayed almost identical twin DGE patterns to healthy controls (Supplementary Figures 2A,B,D and Supplementary Table 1).

To further evaluate whether skin inflammatory diseases could be lined up based on desmosome expression, we performed a gene cluster analysis seeking to partition the PSO, AD, and healthy dataset into groups based on desmosome gene expression. As can be observed in Figure 3E, cluster analysis separated cohort samples into three major branches and seven minor ones. Contrary to expected, PSO and AD samples did not locate in individual major branches; indeed, some PSO and AD samples were grouped with healthy ones. In contrast, the seven minor branches displayed a more cleared clustering where PSO samples were set in two minor ones. Most PSO samples gathered together (71%, 20/28) in one minor 23-sample branch with two additional chronic AD (2/6) and one non-lesional PSO sample leading to 87% of active PSO samples. This first group was characterized by presenting a low expression of DSG4, PKP2, DSG2, DSC1, DSG1, PKP1, and DSP genes while JUP, DSC2, DSG3, and PKP3 genes displayed an increased expression. Secondarily, 17% (5/28) of PSO samples and 19% of AD samples (4/21) set a minor PSO branch consisting of nine total samples without healthy or non-lesional ones. This second cluster was characterized by presenting a low expression of DSG2, DSC3, DSC1, DSG1, PKP1, and DSP while DSC2 and DSG3 were increased. The remaining PSO samples (10%, 3/28) were located in three separate branches, one of them in a 17-sample branch with mostly AD samples (48%, 10/21). As stated above, AD samples were very dispersed, where 48% of AD samples set the most representative branch with non-lesional AD and PSO. However, none of the healthy samples are located in this mostly AD samples branch. In this branch, most desmosome genes were upregulated where DSG3, PKP3, DSC1, DSG1, and PKP1 displayed the highest gene expression. Interestingly, 4/6 of chronic AD with 2/21 AD samples set an exclusive but the smallest cohort branch of 6 total samples. In this chronic AD mostly branch, JUP, DSC2, DSG3, PKP3, DSC1, DSG1, and PKP1 showed a downregulated expression while PKP4 was the only gene with an increased expression. The remaining five AD samples were located with healthy and non-lesional samples in three separate branches. To get a better insight into the distribution and data dispersion of desmosome gene expression in PSO and AD samples, boxplots of mRNA count distributions were performed. As could be seen in Figure 3F, DSG4 displayed the most dispersed gene expression profile between analyzed genes in all lesional and non-lesional groups. Similarly, DSC2 and DSG3 showed a mild gene expression profile. In contrast, most genes displayed a homogeneous profile such as DSC3 and DSG2. DSG-4 was the least expressed desmosome gene of our desmosome transcriptomic analysis where PSO displayed twice fewer counts than healthy and AD. DSG-2 showed a mid-low expression with the lowest counts in PSO as well. Both DSC-2 and DSG-3, which are expressed more than four times in PSO, chronic AD, and AD samples, displayed an intermediate expression (Figure 3F). Desmosome genes with higher read counts displayed similar expression in the cohort. Taken together, we found that both PSO and AD displayed higher expression of two genes, DSG-3 and DSC-2, than healthy samples. On the other hand, DSG-2 and DSG-4 were the lowest expressed desmosome molecules of the cohort in PSO samples (Figure 3F). To our knowledge, this is the first report of DEG between PSO and healthy skin samples taken from RNA-Seq datasets bringing to light the idea that DSG-4 may be considered as a potential PSO gene signature.

## Discussion

The present results support the notion that Dsg-4 deficiency promotes inflammation and alters epidermal integrity during inflammation in an experimental psoriasis-like model. An association between Dsg-4 deficiency and exacerbated psoriasiform dermatitis was demonstrated by an intense leukocyte infiltration in OFA rats in response to IMQ. From human RNA-Seq datasets, we also demonstrated that PSO samples expressed lower DSG4 mRNA levels than healthy samples supporting the association between Dsg-4 levels and inflammatory skin illness. Our results suggest that Dsg-4 adjusts settings on KCs to keep the production of inflammatory genes controlled to support skin homeostasis. To our knowledge, this is the first study to report that the deficiency of Dsg-4 enhances an inflammatory process after TLR stimulation.

Skin DCs, Langerhans cells, and KC subsets express several pathogen recognition receptors; however, we did not explore whether the underlying mechanisms of the enhanced IMQ-induced inflammation in Dsg-4-deficient rats are driven by KCs, DCs, or Langerhans cells (24–26). Similarly, although we could not rule out that Dsg-4 is not crucial for epidermal barrier integrity, a major barrier breakdown was not observed as commensal *S. epidermis* CFU counts were similar in OFA and SD groups. This result agrees with studies that have mostly linked Dsg-4 deficiency with hypotrichosis without reporting a shift in the epidermal barrier (10, 21). Thus, as DCs do not express Dsg-4, we hypothesize that Dsg-4-deficient KCs enhanced the IMQ-induced response, which in turn may prime Langerhans and immune cells by a local amplification loop escalating inflammation with T cell recruitment. Unfortunately, we could not identify additional leukocyte subsets in lesions, but due to the nature of IMQ responses, myeloid and lymphoid cells must be present. We suggest that a suitable Dsg-4 expression in KCs restricts (directly or indirectly) intracellular signaling following an inflammatory stimulation to prevent harmful reactions. Therefore, we propose that Dsg-4 deficiency unleashes KCs intracellular signaling causing the increase of pro-inflammatory chemokines in response to IMQ. Although we have only measured cytokine mRNA levels but not protein loads, it is widely accepted that during acute TLR-NFκB-driven inflammation, early immune response genes rapidly increase their transcription and translation rates (27–29).

The IMQ-induced dermatitis has been established in mice and rats exhibiting similar features of human PSO including thickening of the epidermis, parakeratosis, and inflammatory infiltration (30–33). Although the mice model has been clearly adopted as an IMQ-experimental psoriasis, the rat model has not yet been studied in detail. Indeed, splenomegaly, a psoriasis-related systemic disorder that has been reported in the IMQ-induced mouse model of psoriasis, has not been studied at all in the rat model (34). In contrast to what has been in the mice model, our findings suggest that topical administration of IMQ in SD rats does not lead to spleen or lymph node enlargement. This could be due to different susceptibility to develop T cell-mediated diseases such as observed in experimental autoimmune encephalomyelitis or differences in DC response to IMQ between rodent strains (35, 36). Similarly, OFA rats also showed no signs of spleen disorder despite exacerbated skin inflammation in response to IMQ suggesting that inflammatory events related to Dsg-4 deficiency may not reach distant tissues.

However, similar to the mice model, the outcome of topical IMQ administration on the back skin of rats for 3 days were erythema, scaling, thickening, and inflammation but whether similar mechanisms happen in mice and rats remains unknown (31). Our approach using IMQ-induced inflammatory skin disease on rats does not replicate all features of psoriasis but is nevertheless a pertinent tool to explore the immunological and cutaneous events underlying desmoglein 4 deficiency in rats.

Dsg-4 deficiency would modulate the proliferation and differentiation of KCs in response to IMQ leading to epithelium thickening such as observed here, in PSO and Dsg-1-deficient keratoderma patients as well (37). However, the precise contribution of Dsg-4 to KC differentiation and proliferation remains to be elucidated. Following this line, it has been shown that Smad4, a transcription factor involved in TGF-β-mediated effects such as cell cycling inhibition and immunoregulation, is required for Dsg-4 transcription and, most important, is decreased in PSO (38–40). Although, in our work, Dsg-4 deficiency did not affect TGF-β mRNA levels, TGF-β/IL-17 ratio may favor inflammation. These data could not rule out humoral immunoregulatory factors or a KC–leukocyte contact as commander mechanisms. Additionally, we could not rule out the fact that Dsg-4 deficiency may impair normal KC differentiation, which, in turn, may turn the skin more susceptible to inflammatory reactions. In this line, whether underlying mechanisms of hyper- and parakeratosis observed in OFA rats in response to IMQ are linked to KC differentiation or an immunoregulatory function of Dsg-4 remains to be elucidated. Furthermore, it has been reported that a mutation in Dsg-4 linked to a premature termination results in a rapid progression from proliferative to differentiated KC in the cortex of hair shaft, suggesting that Dsg-4 is tied to epithelial maturation (41).

Our transcriptomic analysis agrees with reported studies where AD and PSO share some immune RNA profiling, to which we contribute to expand this scenario to cadherin family genes, where PSO and AD displayed similar mRNA levels of DSG3 and DSC2 (23). However, whether this unbalanced scenario between Dsgs augments the susceptibility to inflammatory stimuli remains to be elucidated. Surprisingly, there are no reports evaluating any desmoglein isoform expression after an inflammatory stimulation. However, some reports indicate that intestinal epithelial cells enhance their barrier function by selective rearrangement of tight junction molecules after TLR2 stimulation indicating that epithelial activation may trigger a desmosome response (42). A surprising discovery was that PSO samples expressed the lowest Dsg-4 mRNA levels from our desmosome gene analysis in all cohorts. To our knowledge, this is the first time that a decreased DSG4 gene expression is linked to human PSO lesions together with the phenotype data from Dsg-4-deficient rats. Furthermore, no previous data have linked PKP2 expression and PSO, highlighting the need to continue studying desmosome networks.

Although further work must be done, it is remarkable that the lack of Dsg-4 provoked a substantial alteration in the skin response to IMQ, which, in turn, may be associated with risk factors in skin diseases. The clinical importance of our data resides mainly on the potential use of Dsg-4 as a biomarker for screening, diagnostic, prognostic, and monitoring immune-mediated skin diseases such as psoriasis and several dermatitis-like illnesses.

## Materials and Methods

### Animals

Eight- to 12-week-old female OFA rats, which lack body hair as adults and have an intragenic deletion of the Dsg-4 locus in SD genetic background (originally purchased from Iffa Credo, Oncins, France, denominated IFL Nu at that time, subsequently OFA hr/hr), and SD rats bred in our laboratory were used (21). All animals were housed and bred in the specific pathogen-free research animal facility of our institution (Instituto de Medicina y Biología Experimental de Cuyo, Mendoza, Argentina). They were kept in standard conditions, with barriers and controlled light cycle and temperature. Water and food were provided *ad libitum*. All animals were cared in accordance with the Guiding Principles in the Care and Use of Animals of the National Institutes of Health. All procedures were approved by the Institutional Animal Care and Use Committee of the School of Medical Science, Universidad Nacional de Cuyo, 88/2016.

### Experimental Design and Treatment

Animals were distributed randomly into four groups: untreated SD, IMQ SD, untreated OFA, and IMQ OFA. Rats were topically treated with 100 mg Miquimod® cream containing 5% of IMQ (Miquimod®, Lazar, Argentina) once a day for four consecutive days (days 0 to 3) in 4-cm^2^ skin dorsal–lumbar region. Prior to IMQ administration, SD rats were shaved to free the skin of hair. On the fifth day (day 4), euthanasia was performed using carbon dioxide suffocation method. During the protocol, animals were examined to perform a quantitative dermatological score (0: no alteration; 1: mild; 2: moderate; 3: severe; and 4: very severe) based on erythema, thickness, erosions, and scaling. Also, at euthanasia, we evaluated neovascularization, vascular branching, and vessel enlargement.

### Histological Analysis of Skin Tissue

For histological studies, dorsal skin samples from euthanized 8-week-old female OFA and SD rats were obtained. Then, samples were fixed with 4% buffered formalin, embedded in paraffin blocks, and cut into 3–5-micron sections using a microtome (Leica SM2000R, Germany). Hematoxylin and eosin (H&E) staining was used to visualize the morphological structure of skin. Slides were analyzed using light microscopy (Nikon E200) at X400. A quantitative histological score (0: no alteration; 1: mild; 2: moderate; 3: severe; and 4: very severe) was performed based on hyperkeratosis, parakeratosis, apoptotic keratinocytes, lymphocyte exocytosis, spongiosis, subepidermal blister, lymphocytic and neutrophilic infiltrate, follicular infiltration, vasodilatation, perivascular infiltration, and adipocyte necrosis.

### Flow Cytometry Assays

Skin samples of ~150–200 mg were subjected to mechanical disruption with a 200-μm nylon cell strainer (BD Biosciences, San Jose, CA) in PBS supplemented with 10% fetal bovine serum (FBS) until homogeneous single-cell suspensions were reached. Cells were recovered by centrifugation (5 min at 1,800 rpm) and washed once with PBS (5 min at 1,800 rpm). The cells were then resuspended in PBS/2% FBS and stained with PE-Cy5-conjugated anti-rat CD45 (clone OX-1; isotype mouse IgG1 k, BD Biosciences, San Jose, USA) and FITC-conjugated anti-rat CD3 (clone IF4; isotype mouse IgM, Cedarlane Laboratories, US). After staining, cells were washed twice with PBS (5 min at 1,800 rpm), resuspended in 100 μl of PBS, and acquired in a FACSAria III flow cytometer (BD Biosciences, San Jose, CA) and analyzed using FlowJo 7.6.1. software (Tree Star Inc., Ashland, OR, USA). To discriminate between single cells and doublets or cell debris, events were sequentially gated on SSC-A and FSC-A, FSC-W and FSC-H, and SSC-W and SSC-H, plots (Supplementary Figure 3). Absolute T cell counts were determined using a homemade protocol with 1- to 2-μm bright fluorescent beads.

### Gene Expression Analysis of OFA Rats

RNA from 250- to 300-mg skin samples was purified using Trizol reagent (Invitrogen, US) according to the manufacturer's instructions. The integrity of the isolated total RNA was examined by 1% agarose gel electrophoresis, and the RNA concentration was determined by quantification on JENWAY Genova spectrophotometer. Two grams of RNA from skin samples was reverse transcribed by an RT-PCR reaction using the M-MLV Reverse Transcriptase (Thermo Fisher Scientific). Once the cDNA samples were obtained, aliquots were subjected to a real-time PCR carried out for 40 cycles in a Rotor Gene 6000 Real-Time Thermocycler (Corbett, Qiagen, CA, USA). Real-time quantitation was monitored by measuring the increase in fluorescence caused by the binding of EvaGreen (Biotium, US) dye to double-stranded DNA at the end of each cycle of amplification. The analysis and quantification of the results were carried out using the 2–ΔΔCt method where the gene expression was normalized with the control SD untreated sample and the housekeeping gene for Ribosomal Protein S16 (S16). Primer sequences are given in Supplementary Table 3.

### Microbiological Status

Bacteriological analysis was performed by obtaining swabs from a 2-cm^2^ area of the IMQ-treated or untreated skin using a sterile hyssop dipped in glucose broth. Then, the hyssop was immersed in 1 ml of the same broth culture, and 10 seriated 1:10 dilutions were performed. Five microliters of each dilution was plated on glucose-agar plate by exhaustion. Additionally, samples for mycological analysis to observe the presence of filamentous or yeast fungi in a 1-cm^2^ area were obtained by scraping and direct microscopic examination with a drop of 40% NaOH was performed. Afterward, slides were heated over low heat until bubbles came off and observed under conventional microscopy.

### *In silico* RNA-Seq Gene Expression Analysis

Analyses were performed using R version 3.5.1 (http://www.r-project.org/) in a Windows environment with a computer Intel Core i7 with 16GB of RAM. To perform this study, a gene expression dataset was programmatically downloaded from the publicly available Gene Expression Omnibus database (https://www.ncbi.nlm.nih.gov/geo/query/acc.cgi?acc=GSE121212). From these data, 27 were atopic dermatitis (AD) skin samples, 28 were psoriasis (PSO) skin samples, and 38 were healthy skin controls. From PSO and AD patients, lesioned and non-lesioned skin samples were taken, and from AD samples, three belong to chronic lesions. For RNA-Seq expression profiling and differential gene expression (DGE) analysis, low expression counts were filtered out and Voom normalization from the R limma package was performed, allowing normal linear modeling of the RNA counts (43).

On the previously obtained expression matrix, 13 genes that are part of the desmosome were used (DSG1, DSG2, DSG3, DSG4, DSC1, DSC2, DSC3, JUP, PKP1, PKP2, PKP3, PKP4, and DSP). The rows and columns were sorted based on a hierarchical cluster with average linkage and Pearson's correlation distance. According to Silhouette dendrograms, analysis samples and genes were grouped in different clusters.

### DGE Analysis

DGE analysis was performed using the R limma package comparing AD Lesions vs. Healthy samples, AD Non-Lesions vs. Healthy samples, PSO Lesions vs. Healthy samples, PSO Non-lesions vs. Healthy samples, AD Chronic Lesions vs. Healthy samples, AD Non-Chronic Lesions vs. Healthy samples, and, finally, PSO Lesions vs. AD Lesions samples. In all cases, Log_2_ Fold change values were obtained associated with exact *P*-values.

### Statistics

Data and statistical analyses were performed using GraphPad Prism 5 software (GraphPad Software, Inc., San Diego, CA, USA) using one- or two-way analysis of variance (ANOVA) followed by the Bonferroni test. Kolmogorov-Smirnov test was applied to check that the datasets display Gaussian distribution. Kruskal–Wallis test was applied when values did not exhibit Gaussian distribution. For comparison of two means only, Student's *t*-test was used. *P*-values below 0.05 (*P* < 0.05) were considered statistically significant. Data are presented as mean ± SEM, or median with range in RNA expression assays. All *n* values refer to the number of individual rats that are biological replicates.

## Data Availability Statement

The raw data supporting the conclusions of this article will be made available by the authors, without undue reservation.

## Ethics Statement

The animal study was reviewed and approved by Institutional Animal Care and Use Committee of the School of Medical Science, Universidad Nacional de Cuyo, 88/2016.

## Author Contributions

TM-S and MS performed *in vivo* assays and wrote the manuscript in equal contribution. EP and AI performed histology studies. JF-M and FZ performed *in silico* analysis of RNA-Seq datasets. FN performed qPCR assays. MG and DC performed microbiological studies. SV and GJ contributed to the study design and data analysis, proofread the manuscript, and corrected language use. JM-O played a lead role in experimental design, performed *in vivo* assays, wrote the manuscript, and corrected language use. All authors revised and approved the manuscript.

## Conflict of Interest

The authors declare that the research was conducted in the absence of any commercial or financial relationships that could be construed as a potential conflict of interest.

## Supplementary Material

Supplementary Figure 1 Local microbiological status under IMQ administration. **(A)** Bacterial load of *S. epidermis* in the IMQ-treated zones was quantified by culturing on glucose-agar plate by exhaustion. The colony count was calculated according to the following formula: Colony Forming Units CFU/ml= (1,000/0.005)^*^dilution factor. **(B–E)** Representative images from fresh direct microscopic examination that were performed on day 4 (after 4 days of IMQ treatment) in untreated skin from SD **(B)** and OFA **(C)**; and IMQ-treated skin from SD **(D)** and OFA **(E)** rats.

Supplementary Figure 2 Volcano plots of differential gene expression (DGE) analysis achieved by *limma* R method. In the x-axis the log_2_ fold change of **(A)** PSO non-lesion vs. Healthy skin; **(B)** AD non-lesion vs. Healthy skin; **(C)** AD chronic lesion vs. Healthy skin; **(D)** AD non-chronic lesion Vs. Healthy skin. In the *y*-axis, the –log_10_ (adjust *P*-value) is shown (the higher values show smaller adjust *P*-value). Genes with adjust *P* < 0.05 are indicated above the horizontal dashed line.

Supplementary Figure 3 Gating strategy for FACS analysis. Single cells were discriminated from doublets or cell debris by sequentially gating on SSC-A and FSC-A, FSC-W and FSC-H, SSC-W and SSC-H plots.

Supplementary Table 1 Desmosome genes and DGE analysis.

Supplementary Table 2 DGE data from non-lesional and AD chronic lesion samples.

Supplementary Table 3 Primer sequences used in gene expression analysis.

## Abbreviations

AD: atopic dermatitis
Dsg: desmoglein
IMQ: imiquimod
KC: keratinocyte
OFA: Oncin's France Colony A
PSO: psoriasis patients.
