# Supplementary figures and images for: Desmoglein-4 Deficiency Exacerbates Psoriasiform Dermatitis in Rats While Psoriasis Patients Displayed a Decreased Gene Expression of DSG4
Source: Front Immunol. 2021 Apr 29;12:625617. doi: 10.3389/fimmu.2021.625617 (PMC8116535; doi:10.3389/fimmu.2021.625617)

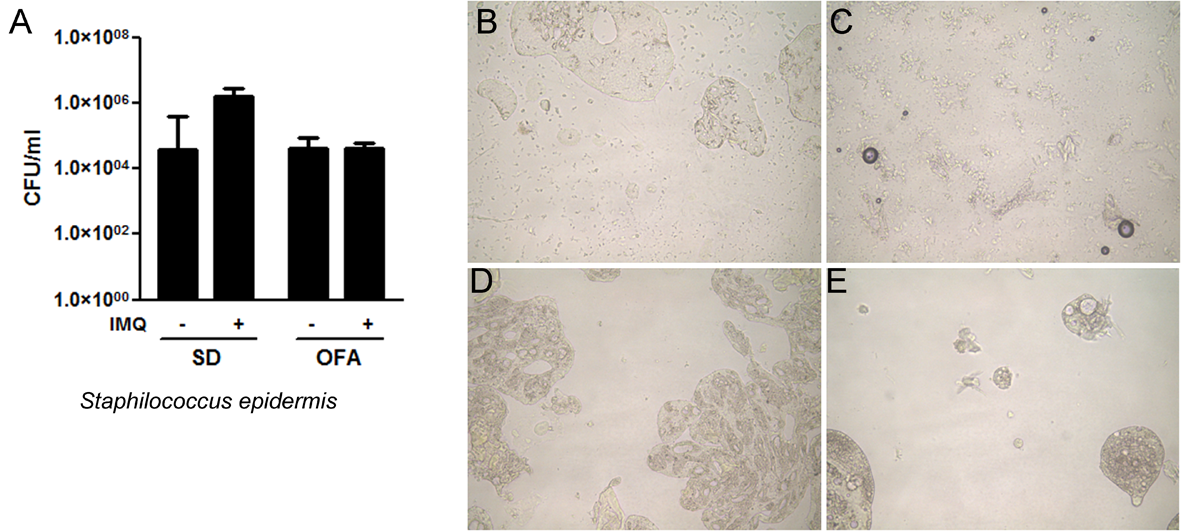

Supplement: Supplementary Figure 1 — Local microbiological status under IMQ administration. (A) Bacterial load of S. epidermis in the IMQ-treated zones was quantified by culturing on glucose-agar plate by exhaustion. The colony count was calculated according to the following formula: Colony Forming Units CFU/ml= (1,000/0.005)*dilution factor. (B–E) Representative images from fresh direct microscopic examination that were performed on day 4 (after 4 days of IMQ treatment) in untreated skin from SD (B) and OFA (C); and IMQ-treated skin from SD (D) and OFA (E) rats. [file Image_1.TIF]

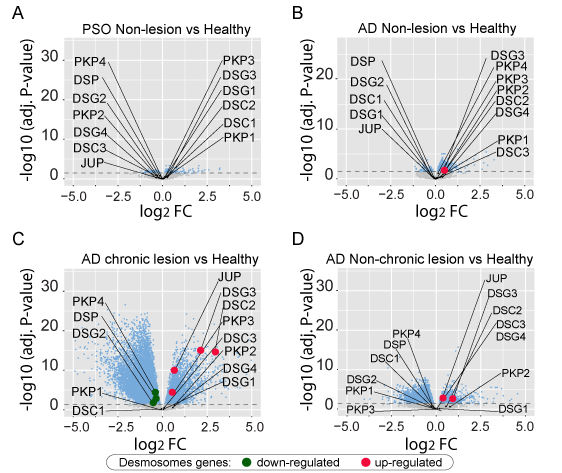

Supplement: Supplementary Figure 2 — Volcano plots of differential gene expression (DGE) analysis achieved by limma R method. In the x-axis the log2 fold change of (A) PSO non-lesion vs. Healthy skin; (B) AD non-lesion vs. Healthy skin; (C) AD chronic lesion vs. Healthy skin; (D) AD non-chronic lesion Vs. Healthy skin. In the y-axis, the –log10 (adjust P-value) is shown (the higher values show smaller adjust P-value). Genes with adjust P < 0.05 are indicated above the horizontal dashed line. [file Image_2.TIF]

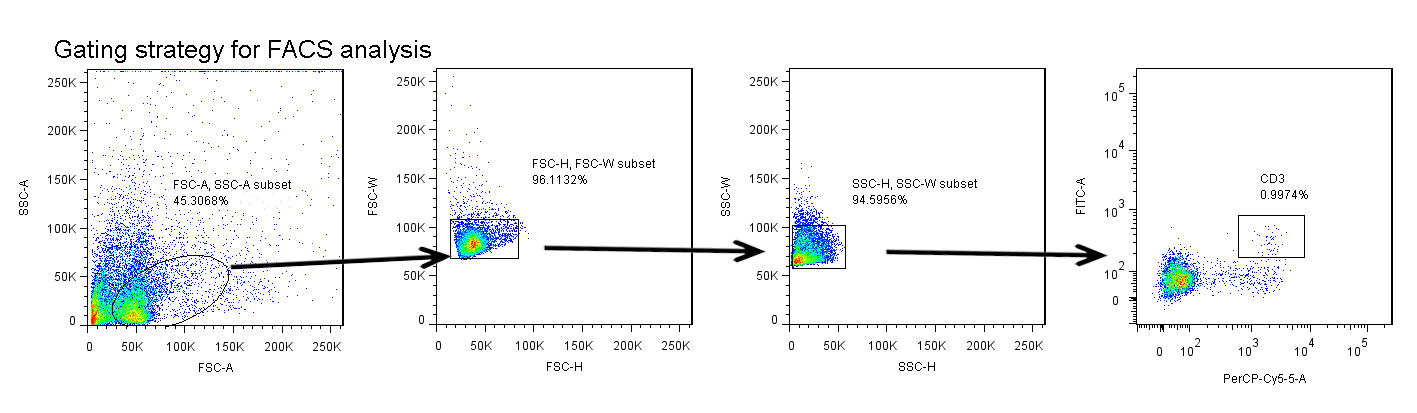

Supplement: Supplementary Figure 3 — Gating strategy for FACS analysis. Single cells were discriminated from doublets or cell debris by sequentially gating on SSC-A and FSC-A, FSC-W and FSC-H, SSC-W and SSC-H plots. [file Image_3.TIF]

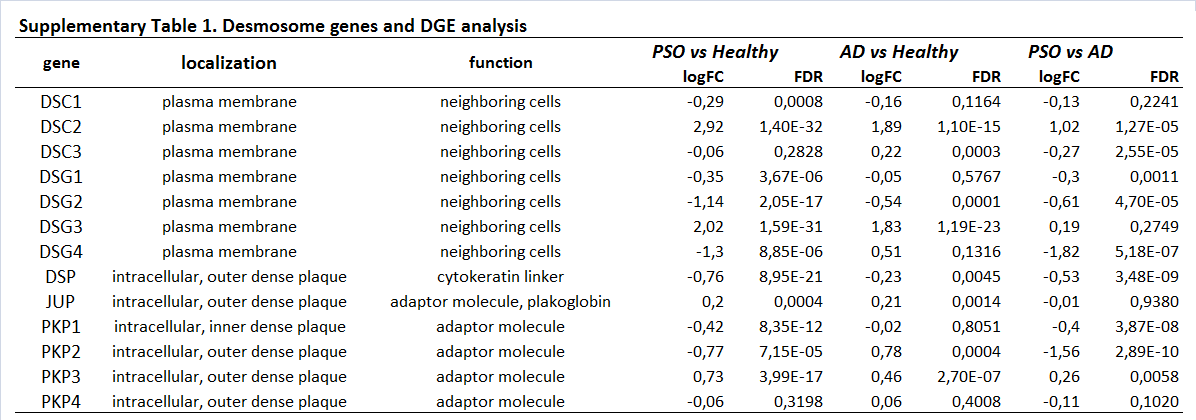

Supplement: Supplementary Table 1 — Desmosome genes and DGE analysis. [file Data_Sheet_1.zip › Image 4 (20).TIF]

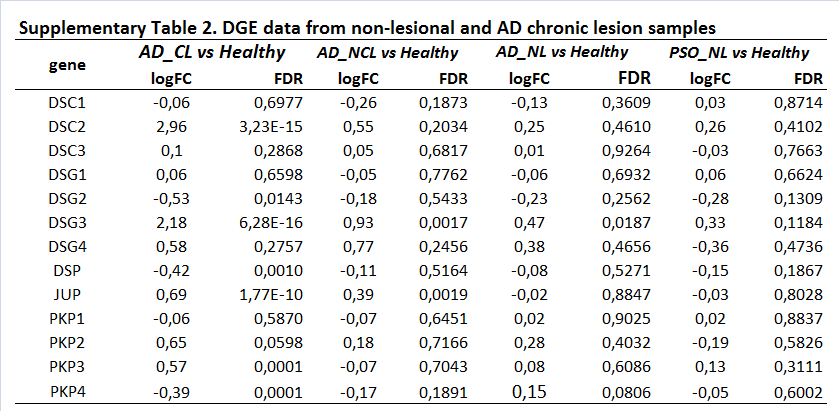

Supplement: Supplementary Table 1 — Desmosome genes and DGE analysis. [file Data_Sheet_1.zip › Image 5 (14).TIF]

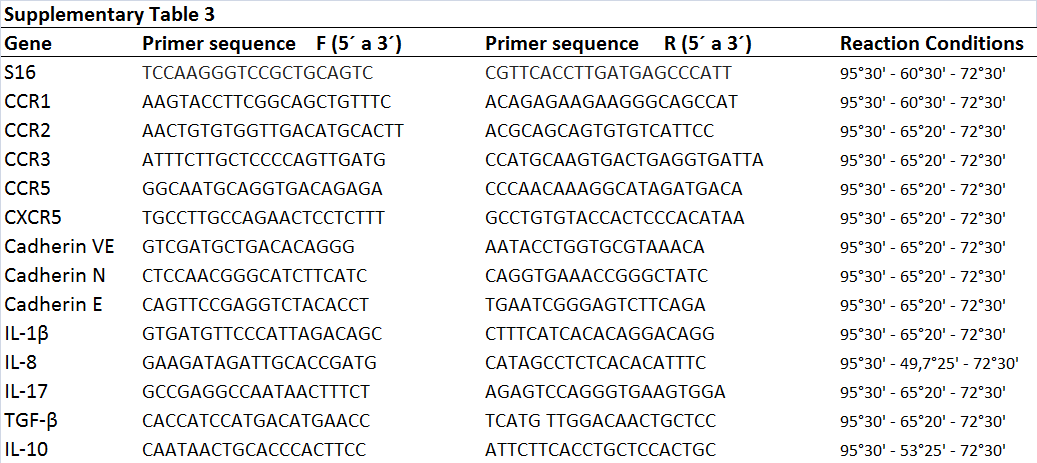

Supplement: Supplementary Table 1 — Desmosome genes and DGE analysis. [file Data_Sheet_1.zip › Image 6 (9).TIF]
